# The early origins of human charity: developmental changes in preschoolers’ sharing with poor and wealthy individuals

**DOI:** 10.3389/fpsyg.2014.00344

**Published:** 2014-06-10

**Authors:** Markus Paulus

**Affiliations:** Developmental Psychology, Ludwig Maximilian University of MunichMunich, Germany

**Keywords:** prosocial behavior, sharing, cognitive development, preschoolers

## Abstract

Recent studies have provided evidence that young children already engage in sharing behavior. The underlying social-cognitive mechanisms, however, are still under debate. In particular, it is unclear whether or not young children’s sharing is motivated by an appreciation of others’ wealth. Manipulating the material needs of recipients in a sharing task (Experiment 1) and a resource allocation task (Experiment 2), we show that 5- but not 3-year-old children share more with poor than wealthy individuals. The 3-year-old children even showed a tendency to behave less selfishly towards the rich, yet not the poor recipient. This suggests that very early instances of sharing behavior are not motivated by a consideration of others’ material needs. Moreover, the results show that 5-year-old children were rather inclined to give more to the poor individual than distributing the resources equally, demonstrating that their wish to support the poor overruled the otherwise very prominent inclination to share resources equally. This indicates that charity has strong developmental roots in preschool children.

## INTRODUCTION

A fundamental principle of humanity and justice reasoning concerns charity (i.e., sharing with the poor, who are in need, but not entitled to resources). Indeed the principle of charity plays an important role in ethical considerations of many religions (e.g., the idea of *Caritas* in Christianity or the *Zakat* as one of five pillars of Islam) and moral philosophy (e.g., Aristotle, 2011). Notwithstanding the fundamental nature of charity for human life, it is largely unknown whether human charity has roots in early development or is a product of an extended period of socialization and enculturation – although such knowledge would be highly informative for recent debates on the nature of human prosociality in social and comparative psychology (Hertel et al., 2002; Tyler, 2003; Penner et al., 2005; Dovidio et al., 2006; Chudek and Henrich, 2011; Paulus and Moore, 2012; Forgas and Tan, 2013; Tomasello and Vaish, 2013; cf. Paulus, 2014).

Recent findings have provided evidence that already preschool children engage in sharing behavior and that a variety of factors affect their sharing decisions. Amongst others, it has been demonstrated that the type of cues uttered by the helpee (Svetlova et al., 2010), the costs associated with sharing (Moore, 2009; House et al., 2013) a shared collaborative history (Hamann et al., 2011; Warneken et al., 2011), and the social relationship between the helper and recipient (e.g., Birch and Billman, 1986; Moore, 2009) play a role in preschoolers’ sharing. For example, Paulus and Moore (2014) gave 3- to 5-year-old children the possibility to share with a friend and with a disliked peer (*Self task*). In a second task (*Other task*), they presented them with another protagonist as well as his friend and a disliked peer (represented by toy bears), and asked to children to predict the protagonist’s sharing decisions. The results showed that the 4- and 5-, but not the 3-year-old children shared more with the friend than with the disliked peer; and also expected another agent to share more with a friend than with a disliked peer. This suggests that early sharing behavior becomes more selective in the course of the preschool years (cf. Hay and Cook, 2007). Yet, it remains an open question whether or not early sharing is actually directed at the other’s material needs, that is, whether or not preschoolers’ take the relative distribution of wealth into account – and share more with poor than with rich recipients. Knowledge about children’s considerations of others’ needs in their sharing would speak to the mechanisms and motivational basis of early sharing and prosocial behavior, which has remained subject to vivid discussion (e.g., Hay and Cook, 2007; Fehr et al., 2008; Jaeggi et al., 2010; Kärtner and Keller, 2012; Chernyak and Kushnir, 2013; Fawcett and Gredebäck, 2013; Kenward and Gredebäck, 2013).

Classical (Damon, 1977) and recent (Kienbaum and Wilkening, 2009; Shaw and Olson, 2013) interview studies with older children suggested that it is during school-age that children (learn to) take others’ needs in resource distribution scenarios into account. For example, Kienbaum and Wilkening (2009) showed that primary school children mainly considered others’ needs when allocating resources to different recipients. Yet, distribution scenarios differ from sharing tasks as in these scenarios children never appear as potential recipients themselves. Accordingly, tasks using sharing paradigms and resource distribution scenarios have partly yielded different results (cf. Olson and Spelke, 2008; Paulus and Moore, 2014). Moreover, it is possible that the interview measures might underestimate children’s actual behavior. First evidence comes from recent studies. McCrink et al. (2010) asked 4- and 5-year-old children as well as adults to evaluate the kindness of different puppets who distributed resources. The authors manipulated the relative wealth of the puppets and the proportion of resources given away. The results indicated that the 4-year-old children’s judgments were only based on the absolute amount of resources given, whereas the 5-year-old children started to take proportions into account (see also Ng et al., 2011). Moreover, a recent study by Paulus et al. (2013a) provided evidence that 5-, but not 3-year-old children include third parties into dyadic sharing situations to a greater extent when these third party individuals possess the majority of resources compared to a situation in which the child himself was the richest person in the triadic situation. This indicates an appreciation of relative wealth as well as fairness at 5, but not 3 years of age. Yet, this procedure relied on a rather demanding measure – active involvement of a third party – and younger children’s failure to do so could be attributed to a number of causes besides a lacking appreciation of wealth and fairness, for example the lacking capacity to simultaneously compare the relative wealth of three individuals. More importantly, as these studies examined reasoning about relative contributions (McCrink et al., 2010) or children’s appreciation of their *own* wealth and the related obligation to share or not (Paulus et al., 2013a), they do not answer the question whether preschoolers’ sharing is at all affected by the *other’s* material needs. Thus, empirical research is needed that directly examines whether preschool children share more with poor than wealthy others.

From a theoretical point of view one could construct two different hypotheses. On the one hand, one could consider findings that preschoolers display sympathy toward others in distress (e.g., Kienbaum et al., 2001; Decety and Svetlova, 2012). This could indicate that from early on children consider others’ needs (e.g., Hoffman, 2000). Accordingly, we would expect that from early on young children share more with needy than wealthy others.

Yet, on the other hand, it is possible that these empathic reactions are largely based on automatic and involuntary affect sharing due to perception-action links (e.g., Preston and de Waal, 2002; de Waal, 2008) and thus do not necessarily involve a consideration of others’ material needs. Moreover, recent studies have provided evidence for a dissociation between the different varieties of prosocial action (e.g., Dunfield et al., 2011; Dunfield and Kuhlmeier, 2013; Paulus et al., 2013b), indicating that the processes that underlie empathy-motivated comforting might not be related to early sharing at all.

Importantly, recent findings demonstrated that even in sharing situations that bear no cost to the child, 2-year-old children do not understand others’ material needs and do not support the other, unless the other explicitly shows his wish (Brownell et al., 2009). Moreover, research by Blake and Rand (2010) has provided strong evidence that in sharing situations even a majority of 3-year-old children do not share with another person, whereas they do so only by the age of 5. Thus, based on this line of reasoning a second hypothesis could assume that early sharing may not be motivated by a consideration of others’ material needs and by a wish to support the poor. In contrast, early sharing could be based on motivations that are independent of the others’ material wealth – for example, a motivation to interact with another person (i.e., a social, yet not genuinely prosocial motivation; Paulus, 2014) as sharing with others helps to establish social contacts (e.g., Binmore, 2006); or a motivation to comply with another’s request (e.g., Brownell et al., 2009; Dunfield et al., 2011) – and consequently young children would not share more with poor than wealthy others based on a consideration about their needs.

Taken together, it remains an open question whether or not early sharing is motivated by a genuine appreciation of others’ material needs and relative wealth; and thus a motivation to allocate more resources to poor than to rich agents. If children’s sharing behavior is based on an evaluation of others’ relative wealth, then they should share more with poor than wealthy individuals. Thus, when are children’s sharing behavior based on an evaluation of the recipients’ material needs?

Given the fundamental role of charity for humanity and moral behavior, the present study was designed to examine the early origins of human charity. As our main interest to examine the factors and mechanisms subserving sharing behavior, Experiment 1 employed a sharing task to examine whether preschool children take others’ indigence into account when sharing resources with others. Experiment 2 relied on a resource allocation paradigm to investigate children’s inclination to distribute resources between poor and rich individuals. As previous work using a variety of different measures has pointed to significant developmental changes in children’s sharing behavior in the course of the preschool period (e.g., Blake and Rand, 2010; Paulus et al., 2013a), we choose to examine 3- and 5-year-old children.

## EXPERIMENT 1

The current study aimed at clarifying whether young children consider others’ material needs in their sharing behavior. As a consequence, Experiment 1 employed a sharing task to assess preschoolers’ sharing with poor and wealthy recipients. To keep our results comparable to previous findings, we used a sharing task modeled on previous research (Fehr et al., 2008; Olson and Spelke, 2008; Moore, 2009). It consisted of several situations in which the child could share stickers with one of two different recipients; an agent who had a sticker book full with stickers (rich agent) and an agent who barely had any stickers (poor agent). Two choice types were included. In the even choice type – associated with low costs for the child – the child could choose between two stickers for herself and two for the other (2/2), or three for herself and one for the other (3/1). In the uneven choice type – associated with high costs for the children – the child could choose between three stickers for herself and one for the other (3/1), or one for herself and three for the other (1/3). Previous research has successfully employed similar amounts of resources in 3-year-old children (Olson and Spelke, 2008). We included these two different choice types as they both assessed whether the child would be willing to sacrifice own resources to support another person and as a comparison between the two types would clarify whether the costs associated with sharing would interact with a potential inclination to share more with poor than rich people (e.g., when the cost is quite high as in the uneven trials children would show low sharing and no differentiation, in cases of lower costs as in the even trials differential sharing would become evident).

### METHOD

#### Participants

The sample included 17 3-year-old children (*M* = 42 months, SD = 1.7; seven boys) and 17 5-year-old children (*M* = 65 months, SD = 3.7; six boys). All participants were typically developing children from a larger European city and were of mixed socioeconomic status. Informed consent for participation was given by the children’s caregivers. The study followed the ethical principals outlined by the Helsinki’s 1964 declaration and the recommendations of the German Psychological Society.

#### Materials

Materials included colored stickers, which have been successfully used in previous studies (e.g., Prencipe and Zelazo, 2005; Gummerum et al., 2010), an envelope for the child and two sticker books for the two recipients. The sticker book of the poor agent contained ca. three stickers, whereas the sticker book of the wealthy agent contained around 50 stickers. We choose to employ this large difference to prevent the poor agent to become richer than the wealthy agent in the course of the task. Two toy figures (toy bears; appr. 30 cm high) served as possible recipients. Previous studies have successfully employed animal characters or toy figures to investigate children’s reasoning about social situations and resource distributions (e.g., Fawcett and Markson, 2010; McCrink et al., 2010; Kenward and Dahl, 2011; Kanngiesser and Warneken, 2012). Moreover, Paulus and Moore (2014) found no difference in children’s decisions when toy bears were involved to represent a sharing situation between friends or disliked agents, or when children were asked to share stickers with a friend or a disliked peer.

#### Procedure

Children were tested individually in a quiet room. Experimental sessions were scored online by the experimenter and videotaped for later reliability coding.

The color of the bears’ shirts served as their names during the entire experimental session. The participants were familiarized with the recipients. In particular, they were told that both bears love stickers and that they like to collect them. Subsequently, the experimenter showed the child that one of the bears (rich agent) had already a lot of stickers (the sticker book full of stickers), whereas the other one had barely any stickers (the sticker book containing only three stickers; poor agent). Importantly, the experimenter described both agents and their possessions in the same neutral manner, to not induce sympathy for the poor agent (and thus bias children’s decisions) by means of her verbal intonation. After the presentation of the agents, the experimenter introduced the task. She explained that the child could choose items for both herself and another bear. The items chosen for the bears would be handed over to them and kept in a bowl; the items kept by the child would be collected and could be taken home by the children in their envelope.

Children were then presented with three blocks of trials. Each block contained one trial of each of four trial types. The trial types resulted out of the factorial combination of the factor Sharing Partner (Rich agent, Poor agent) and the factor Choice Type (even, uneven). Trial order and the order of the choices offered in each question were counterbalanced among blocks and participants.

The protocol followed the studies by Moore (2009), Paulus and Moore (2014). In every trial, the experimenter put the respective number of items on the table and demonstrated the options by dividing the stickers in the respective manner and by pretending to move the stickers to the respective recipients. This part of the protocol ensured that the options were not only presented verbally, but also concretely experienceable.

After the presentation of the agents and again after they completed the task, participants were asked to identify the agent who has a lot of stickers and the agent who has only few stickers. Data from 27 participants were obtained in this manipulation check (due to experimenter mistake, seven children were forgotten to be asked). All of these correctly identified the respective agents.

#### Data analysis

Data were coded by the experimenter. For each trial, participants received a score of 1 if they chose the option that afforded relatively more items to the respective recipient than to themselves. That is, they received a score of 1 when they choose the (2/2) option in the even trials and the (1/3) option in the uneven trials. Scores were recorded as proportional measures of equitable choices for each trial type. 12 randomly chosen children (35%) were recoded by a second person blind to the purpose of the study. Both raters agreed to 100%.

Experiment 1 examined whether children were more inclined to share when they were paired with a poor than with a wealthy recipient. In other words, we were interested whether the factor representing recipient’s wealth affected children’s sharing. Thus, the main test was a 2 (Age Group: 3, 5) × 2 (Recipient: Rich agent, Poor agent) × 2 (Trials: even, uneven) mixed-model repeated measures analysis of variance (ANOVA).

### RESULTS

**FIGURE 1:**
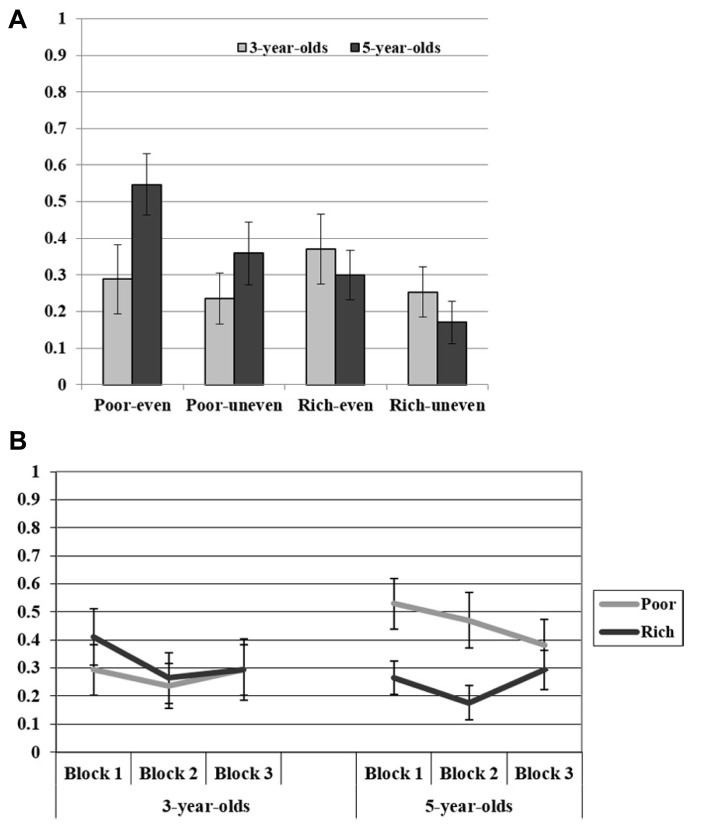
Panel **(A)** shows the mean proportion of trials on which participants choose the option that afforded relatively more items to the respective recipient in Experiment 1. Panel **(B)** shows the mean proportion of trials (averaged across trial types) per block in Experiment 1. Error bars indicate standard errors of the means.

Descriptive results are shown in **Figure 1A**. The ANOVA revealed a main effect of Trial, *F*(1,32) = 10.915, *p* < 0.01, η ^2^ = 0.25. This shows that the 3- and 5-year-old children chose the option that was more beneficial for the respective recipient more often in the even choice trials than in the uneven choice trials, suggesting that children were more generous when it was less costly for them. Importantly, the analysis revealed also an interaction effect of Recipient and Age, *F*(1,32) = 6.071, *p* < 0.05, η^2^ = 0.16. To follow up on the interaction between Recipient and Age, we conducted *post hoc t*-tests for each age group, comparing whether children afforded more resources to the poor than to the wealthy recipient. These analyses showed that the 5-year-old children shared more with the poor (*M* = 0.45, SE = 0.08) than the wealthy recipient (*M* = 0.24, SE = 0.04), *t*(16) = 2.218, *p* < 0.05. This was not the case for the 3-year-old children, *t*(16) = 1.074, *p* = 0.30, who did not share more with the poor (*M* = 0.26, SE = 0.07) than the wealthy recipient (*M* = 0.31, SE = 0.07).

Next, we compared children’s performances in the different trial types against chance by means of *t*-tests (with behaviors below chance indicating a primarily selfish motive). These analyses showed that for the 3-year-old children performance in all trial types was below chance (all *p*s < 0.05), except for the even-rich trials, *t*(16) = 1.351, *p* = 0.20. In the 5-year-old children, all trial types involving the rich agent were below chance (all *p*s < 0.01), whereas both trial types involving the poor agent were not different from chance, *t*(16) = 0.563, *p* = 0.58 for the even-poor trials, and *t*(16) = 1.638, *p* = 0.12 for the uneven-poor trials, respectively.

As we were interested whether there were general changes in children’s performance over time (e.g., indicating that even the 3-year-old children showed some preference for the poor at the beginning of the experiment), we additionally compared performance across blocks (see **Figure 1B**). Given that the previous analysis did not reveal an interaction effect with respect to trial type (i.e., trial type was orthogonal with respect to the age and recipient), we averaged for every child the data for each block and recipient across both trial types. Thus, we calculated for every participant and for each block, how well he/she treated the poor and the wealthy recipient. A 2 (Age Group: 3, 5) × 4 (Blocks: 1, 2, 3) × 2 (Recipient: Recipient: Rich agent, Poor agent) mixed-model repeated measures ANOVA yielded only a significant interaction effect between Recipient and Age-Group, *F*(1,32) = 6.069, *p* < 0.05, η^2^ = 0.16 (all other *p*s > 0.13), replicating the previously reported effect that the 5-year-old, but not the 3-year-old children treated the poor recipient better than the rich recipient.

### DISCUSSION

Experiment 1 was designed to examine whether children take others’ needs in their sharing behavior into account. The results provide evidence that preschool children as young as 5 years of age share more with poor than wealthy individuals. Furthermore, the results show a strong developmental effect as 3-year-old children’s sharing behavior was largely not affected by the others’ wealth. Also a follow-up analysis on changes over time (i.e., experimental blocks) did not reveal any effect, excluding the possibility that an initially existing preference for the poor recipient in the 3-year-old children became weaker in the course of the study and did therefore not reach significance. These results suggest that humans’ inclination to follow the principle of charity develops in the preschool period.

Note that children of both age-groups showed a tendency to bias their choices toward themselves (i.e., choosing the option that afforded more items to the other below 50%). These results are partly in line with previous findings on young children’s sharing behavior (e.g., Blake and Rand, 2010; Smith et al., 2013). This demonstrates that all children understood the task, acted strategically, and supports thus the validity of our method.

Most importantly, for the 5-year-old children this was not the case when being confronted with the poor individual. Here, their selfish motivation was decreased and they showed a higher probability of choosing the option that benefitted the other. Interestingly, this pattern was slightly reversed in the 3-year-old children. They showed a decreased tendency to act selfishly in one trial type involving the rich agent. This might suggest the presence of a tendency to favor advantaged and lucky others over disadvantaged others (cf. Olson et al., 2006) already in 3-year-old children.

Yet, it is possible that even younger children at least understand the idea that more needs to be given to poor than wealthy people, but that this understanding is masked in a task in which they have to share their own resources (cf. Olson and Spelke, 2008). That is, it is possible that issues of self-control could interfere with their understanding that they should more with the poor recipient. Support for this point comes from work demonstrating relations between self-control and strategic social behavior (Steinbeis et al., 2012) as well as between inhibitory control and preschool children’s likelihood to share (Aguilar-Pardo et al., 2013). Thus, to investigate the developmental differences in preschooler’s considerations of others’ material needs in greater detail, we therefore conducted Experiment 2. We employed a resource allocation paradigm (cf. Olson and Spelke, 2008; McCrink et al., 2010; Kenward and Dahl, 2011) in which children had to distribute resources between a rich and a poor individual.

Based on previous findings of developmental differences between 3- and 5-year-olds’ inclination to restore fairness in cases of unequal resource distribution (Paulus et al., 2013a) and developmental differences in children’s general inclination to share (Blake and Rand, 2010; Smith et al., 2013) as well as the results of Experiment 1, we expected that the 5-, but not the 3-year-old children would allocate more resources to the poor than the wealthy agent.

## EXPERIMENT 2

In Experiment 2, children could distribute stickers between the same two recipients as in Experiment 1. Three different choice types were included. In the uneven choice type, the child could choose between three stickers for the poor agent and one sticker for the rich agent (3/1) or one sticker for the poor agent and three stickers for the rich agent (1/3), both choices urging the child to prefer one agent over the other. In the even-poor choice type, the child could choose between two stickers for each recipient (2/2) or three stickers for the poor agent and one sticker for the rich agent (3/1). This choice type investigated in particular whether children preferred to share equally or to follow the principle of charity. In the even-rich choice type, the child could choose between two stickers for each recipient (2/2) or one sticker for the poor agent and three stickers for the rich agent (1/3). This choice type controlled for a preference for the poor agent in the even-poor trials was not merely motivated by a preference for giving someone a large amount of resources.

### METHOD

#### Participants

The sample included another group of 17 3-year-old children (*M* = 42 months, SD = 1.3; eight boys) and another 16 5-year-old children (*M* = 67 months, SD = 1.3; eight boys). Sample characteristics and consent protocol were the same as in Experiment 1.

#### Materials and procedure

The procedure closely followed Experiment 1 with the following difference. Children were presented with four blocks of trials. Each block contained one trial of each of three trial types (uneven, even-poor, even-rich). Trial order, as well as the order of the choices offered in each question was counterbalanced among blocks and participants. As a prompt, children were asked whether they would like, for example, to choose three stickers for blue bear and one sticker for red bear; or one sticker for blue bear and three stickers for read bear. As in Experiment 1, the option were not only presented verbally, but also physically demonstrated.

Data from 26 participants were obtained in the manipulation check (due to experimenter mistake, seven children were forgotten to be asked). All but one 5-year-old correctly identified the respective agents.

#### Data analysis

Data were coded by the experimenter. For each trial, participants received a score of 1 if they chose the option that afforded relatively more items to the poor recipient. That is, they received a score of 1 when they chose the (3/1) option in the uneven trials, the (3/1) option in the even-poor trials and the (2/2) option in the even-rich trials. Scores were recorded as proportional measures of equitable choices for each trial type. 12 children (35%) were recoded by a second person. Both raters agreed to 98%.

Experiment 2 examined whether children distributed more items to poor than to wealthy recipients. That is, in contrast to Experiment 1 (where the crucial manipulation was realized between trials) we were interested whether within a trial type child showed a preference for one over the other recipient. Consequently, the main analyses were *t*-tests against chance level (50%).

### RESULTS

**FIGURE 2:**
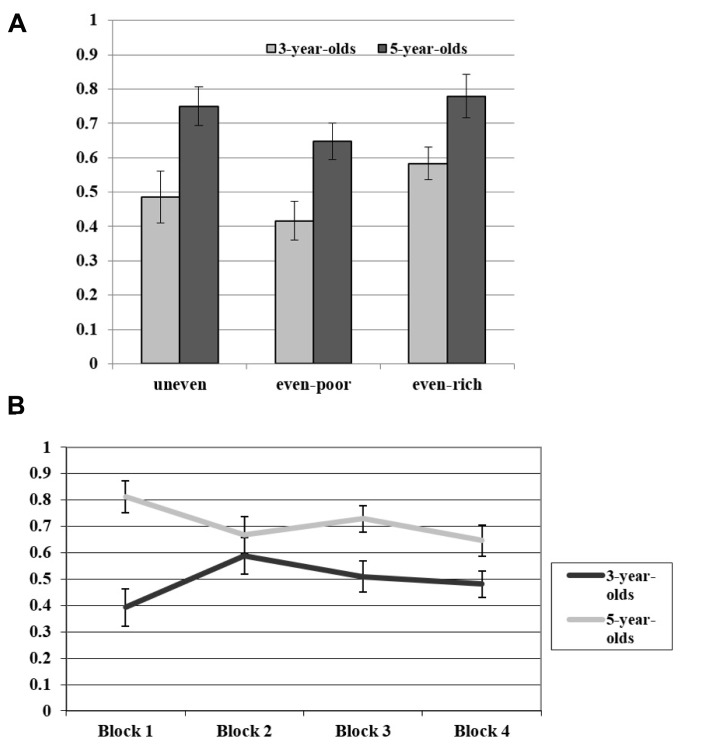
Panel **(A)** shows the mean proportion of trials on which participants chose the option that afforded relatively more items to the poor recipient than to the rich recipient in Experiment 2. Panel **(B)** shows the mean proportion of trials (averaged across trial types) per block in Experiment 2. Error bars indicate standard errors of the means.

Descriptive results are shown in **Figure 2A**. The *t*-tests showed that the 3-year-old children did not show any preference in their choice of resource distribution between the rich and the poor agent, *t*(16) = 0.194, *p* = 0.85, *t*(16) = 1.496, *p* = 0.15, and *t*(16) = 1.772, *p* = 0.10, for the uneven, even-poor, and even-rich trials, respectively. In contrast, the 5-year-old children’s choices yielded a clear pattern as they differed for all trial types from chance, *t*(15) = 4.140, *p* = 0.001, *t*(15) = 2.449, *p* < 0.05, and *t*(15) = 4.000, *p* = 0.001, for the uneven, even-poor, and even-rich trials, respectively.

To further substantiate these findings, we directly compared children’s performance across the trial types. A 2 (Age Group: 3, 5) × 3 (Trial Types: uneven, even-poor, even-rich) mixed-model repeated measures ANOVA yielded a main effect of Age Group, *F*(1,31) = 18.128, *p* < 0.001, η^2^ = 0.37, showing the 5-year-old children afforded more items to the poor recipient (*M* = 0.71, SE = 0.04) than the 3-year-old children (*M* = 0.50, SE = 0.04). Additionally, the analysis revealed a main effect of Trial Type, *F*(2,62) = 3.482, *p* < 0.05, η^2^ = 0.10. There was no effect of the interaction term, *F* < 1. *Post hoc* comparisons for the Trial Types showed that even-poor and even-rich trials differed from each other, *t*(32) = 2.613, *p* < 0.05 (all other *p*’s > 0.10).

As we were interested whether there were general changes in children’s performance over time (e.g., indicating that even the 3-year-old children showed some preference for the poor at the beginning of the experiment), we additionally compared performance across blocks (see **Figure 2B**). Given that the previous analysis did not reveal an interaction effect of age group and trial type (i.e., trial type was orthogonal with respect to the age), we averaged for every child the data for each block over all trials. Thus, we calculated for every participant an average performance value for each block. A 2 (Age Group: 3, 5) × 4 (Blocks: 1, 2, 3, 4) mixed-model repeated measures ANOVA yielded a main effect of age group, *F*(1,31) = 18.498, *p* < 0.001, η^2^ = 0.37, replicating the finding that the 5-year-old children awarded more items to the poor than the 3-year-old children. Additionally, the analysis showed an interaction effect between the factors Age Group and Block, *F*(3,93) = 2.979, *p* < 0.05, η^2^ = 0.09. *Post hoc* independent samples *t*-tests were performed to compare age differences for every block. These analyses showed that the performances of the two age groups differed significantly from each other in the first block, *t*(31) = 4.462, *p* < 0.001, the third block, *t*(31) = 2.576, *p* < 0.05, and the fourth block, *t*(31) = 2.211, *p* < 0.05, but not the second block, *t*(31) = 0.783, *p* = 0.44.

### DISCUSSION

Experiment 2 examined the developmental origins of children’s inclination to allocate more resources to poor than to wealthy individuals in a resource distribution paradigm. The results of Experiment 2 provide clear evidence that the 5-year-old children showed, across three different trial types, a consistent inclination to rather distribute resources to a poor than to a wealthy agent. The 3-year-old children, in contrast, showed no such preference in any of the various trial types. Moreover, an additional analysis revealed no systematic changes over time in this pattern. In sum, corroborating the findings from Experiment 1, the results provide evidence that 5-year-old, but not 3-year-old children take charity considerations into account when deciding of how to allocate resource between different recipients.

A direct comparisons of the trial types with each other showed that children across both age-group choose the option that afforded relatively more items to the poor recipient more often in the even-rich than in the even-poor trial type. What could this mean, particularly given the fact that the 3-year-old children showed no tendency to distribute more stickers to the poor than to the wealthy recipient? Note that in the even-rich trials the option, which was beneficial for the poor, was the equal (2/2) option (instead of distributing 3 to the rich and 1 to the poor). In the even-poor trials, the option, which was beneficial for the poor, was the (1/3) option (i.e., 1 to the rich, 3 to the poor), whereas the equal (2/2) option was less beneficial for the poor. The fact that the 3-year-old children choose the – for the poor recipient more beneficial – (1/3) option in 42% actually shows that they choose the (2/2) option in 58%. In other words, the results indicate a preference for choosing the equal option (2/2) across trial types and across age groups. For the 5-year-old children this tendency interacted with a stronger tendency to support the poor recipient, which is most clearly expressed in the fact that they even in the even-poor rather supported the poor than distributing the resources equally. In contrast, the 3-year-old children had no such tendency to support the poor recipient. Consequently, they only showed a small preference for the equal option, which presented itself either as a positive or negative deviation from chance level, depending on whether the equal option was beneficial for the poor or the rich. This finding thereby confirms previous findings demonstrating weak preferences for equal distributions in young preschool children. Shaw and Olson (2012) provided evidence that school-aged, but not younger children favor equal distributions. House et al. (2012) reported that younger preschoolers are rather inclined to provide benefits to others than to choose egalitarian outcomes. Finally, Paulus et al. (2013a) demonstrated an impact of own wealth on third party involvement only in 5-, but not 3-year-old children.

More interesting, however, is the finding that the 5-year-old children were rather inclined to support the poor recipient than distributing the resources equally between both recipients. The consequences will be discussed in the next section.

## GENERAL DISCUSSION

According to the principle of charity, scarce resources should be distributed considering the relative indigence of the recipients. Such considerations of others’ neediness play a vivid role in religions and philosophical theories on prosociality (e.g., Aristotle, 2011) and are substantial for our concept of humanity. This study aimed at investigating the early roots of human charity in two experiments with 3- and 5-year-old children. The experiments provide converging evidence that preschool children of 5 years of age take others’ indigence into account when sharing resources with different recipients or when allocating resources between recipients. It extends previous findings on school-aged children’s appreciation of others’ material needs (e.g., Kienbaum and Wilkening, 2009), by demonstrating that this tendency develops between 3 and 5 years of age – pointing thus to the early roots of human charity.

From a theoretical point of view, knowledge about the principles guiding children’s sharing and resource allocation behavior informs us about the psychological mechanisms underlying early prosocial behavior. In other words, it would help us understand why humans in general and young children in particular engage in prosocial behaviors (for discussion see Paulus, 2014). The present results show that by at least 5 years of age sharing is motivated by children’s considerations of the others’ wealth. That is, already preschool children rely on the principle of charity when sharing or distributing resources with/to others, suggesting that by this age sharing is motivated by considerations to fulfill others’ material needs.

Importantly, in Experiment 2 the 5-year-old children did not only prefer to give more to the poor than the wealthy individual in the uneven trials, when they were urged to prefer either of the two recipients. They were also rather inclined to give more to the poor individual than distributing the resources equally between the two recipients in the even-poor trials. This shows that charity considerations have strong developmental roots in the preschool age.

Why are the 5-year-old children inclined to hand over more stickers to the person in need than the wealthy person? It is clear that material need and material wealth are relational concepts, i.e., they are relative to the context. That is, although in our study the recipient with only two stickers was indubitable more needy than the other recipient, he would have been more wealthy when the other recipient would have had no stickers at all. It is thus unlikely that a particular personal trait or characteristic of the poor recipient triggered the children’s behavior. Rather, it seems likely that their decision to prefer the needy recipient was based on fairness considerations, i.e., on a motivation to equalize outcomes. This shows that their wish for equal outcomes trumped the otherwise very prominent inclination to share resources equally between partners as suggested by recent findings (e.g., Blake and McAuliffe, 2011; Hamann et al., 2011). That is, our study demonstrates that – next to a tendency for procedural equality during sharing, i.e., giving everyone the same amount – preschool children show a strong inclination for equal outcomes. This suggests that already preschool children are sensitive to aspects of procedural and distributive justice (for an extended discussion see Müller and Kals, 2007).

In contrast, although even the 3-year-old children showed some sharing behavior, it was largely not affected by the others’ material needs. Indeed, if anything, the 3-year-old children showed a tendency to be less selfish toward the rich recipient, suggesting a tendency to favor the lucky (cf. Olson et al., 2006). There are several possible interpretations for the lack of the consideration of others’ needs. First, it is possible that a strong motivation for equal sharing dominated their behavior (even though they might consider others’ needs). Yet, this interpretation is unlikely given that in Experiment 1 the 3-year-olds did not opt for the equal option in the majority of trials. Additionally, in Experiment 2 they showed no preference for the poor even in trials in which there was no equal option (i.e., they were urged to either give more to the poor or the wealthy agent) or when the equal option was at the same time the option that was most beneficial for the poor. Second, one could argue that the employment of toy bears hampered 3-year-old children’s performance. Yet, this interpretation is unlikely given that previous studies have successfully used puppets and toy figures to examine children’s social understanding and choices (e.g., Fawcett and Markson, 2010; Meyer et al., 2010; Kenward and Dahl, 2011). Moreover, Paulus and Moore (2014) found the same developmental pattern in sharing tasks employing toy bears or children’s actual friends and disliked peers as potential recipients, providing a direct empirical validation for the method used in the current study.

As a consequence, we suggest a third interpretation, i.e., that our results indicate that 3-year-olds just do not consider others’ material needs in their sharing behavior, suggesting that these early instances of sharing are not primarily motivated by a consideration of others’ needs, but follow simpler heuristics. This interpretation is supported by the fact that even in the resource allocation paradigm (Experiment 2) children did not allocate more resources to the poor individual. This interpretation relates to other studies that even in situations in which sharing would not be costly, toddlers do not allocate resources to another person without being addressed by the other through explicit cues expressing his needs and wishes (Brownell et al., 2009). In line with this, Dunfield et al. (2011) reported that 2-year-old children indeed gave more crackers to a person who had no crackers (experimental condition) compared to a person who also possessed some (control condition). Yet, the person in the experimental condition (but not in the control condition) explicitly requested items from the child by placing her hand out with the palm facing up. Additionally, she made a sad face. Children’s preferential giving to this person could thus be based on a reaction to the explicit request for items rather than a genuine appreciation of the other’s material need. The current study controlled for these issues, suggesting that the 5-year-olds’ preferential sharing with the poor recipient is based on a genuine appreciation on others’ material needs, which does not seem to be in place in 3-year-old children. If this interpretation were true, the present results point to a fundamental change in the motivations underlying early prosocial action in the course of the preschool period (cf. Hay and Cook, 2007; Paulus, 2014).

How does development then proceed? Interestingly, a recent study by Svetlova (2013) employing a distribution scenario suggests that even younger children show a slight tendency to allocate more resources to poor than to wealthy agents, when the experimenter emotionally cues the situation of the needy recipient. That is, in this study the experimenter modulated her voice in a neutral manner when presenting a wealthy puppet and in a pitiful manner when presenting the poor puppet. In this situation, even 3-year-old’s showed a tendency to support the poor agent. Given that the perception of the experimenter’s emotional tone triggers empathic reactions (cf. Hoffman, 2000; Decety and Svetlova, 2012), it is not unlikely that the children’s responses in this study were supported by the experimenter’s emotional cues, which might have induced sympathy for the poor, but not the rich recipient. This is an important finding as we suggest that such an induction of sympathy could also explain the developmental difference presented in the current study. Whereas 3-year-old children show no spontaneously occurring sympathy with materially needy others (or only after it was externally cued by emotional signals; Svetlova, 2013), 5-year-old children might be better able to put themselves into the shoes of the needy recipient and, as a consequence, showed more sympathy, and thus more prosocial behavior, toward the needy agent; indicating an abstract understanding that poor agents deserve more resources than rich agents. This explanation might be supported by recent findings that early sympathy predicts the development of sharing behavior (Malti et al., 2012) and that mood effects fairness decisions in dictator games (Forgas and Tan, 2013).

The present study is not only informative for current social psychological theories on the nature of prosocial behavior and justice considerations (cf. Tyler, 2003; Penner et al., 2005; Dovidio et al., 2006), it also leads to novel research questions. Our results show that by 5 years of age, children reduce inequality by handing more resources to a poor recipient than a wealthy one. Interestingly, studies with older children provided evidence that under some circumstances people accept inequalities (Almas et al., 2010). It would thus be interesting to examine whether and under which circumstances the 5-year-old children would accept the unequal distribution of resources, without trying to equalize it by providing more resources for the poor recipient. Future research is needed to address this question.

Taken together, the present study shows that a unique characteristic of human moral reasoning, i.e., the principle of charity and distributive justice, has its developmental origins in the preschool period. That is, considerations of charity develop at an age long before humans engage in theoretical debates on the fairest manner of distributing scarce resources as evident in religious prescriptions and philosophical theories.

## Conflict of Interest Statement

The author declares that the research was conducted in the absence of any commercial or financial relationships that could be construed as a potential conflict of interest.
